# Knowledge, Attitude, and Adaptation to Climate Change in Ghana

**DOI:** 10.1155/2020/3167317

**Published:** 2020-11-25

**Authors:** Stephen T. Odonkor, Emmanuel Nene Dei, Anthony M. Sallar

**Affiliations:** ^1^School of Public Service and Governance, Ghana Institute of Management and Public Administration, Accra, Ghana; ^2^National Blood Services, Accra, Ghana; ^3^School of Liberal and Social Sciences, Ghana Institute of Management and Public Administration, Accra, Ghana

## Abstract

Climate change is a serious challenge to human existence. It threatens efforts towards the attainment of sustainable development goals and aggravates conditions that lead to health inequities and inequalities for vulnerable populations. The study aimed to investigate knowledge and adaptation to climate change among people in Ghana. A nationally representative survey of Ghanaian adults (*N* = 674) was conducted from August 1, 2019, to December 31, 2019. Results showed that 43.9% of the respondents understood the meaning of climate change. Respondents perceived the causes of climate change to include burning of fossil fuel, deforestation, natural events such as ocean currents, carbon emission from vehicles and industries, agricultural emissions of nitrous oxide from fertilizers, and an act of God. About 53.1% and 41% of the male and female respondents, respectively, had an encounter with climate change-induced natural disasters. About two out of five respondents (43%) were either afraid or confused about climate change. Distilled or maintained public drainage from waste (30.2%) and clearing drains (25.6%) was the leading adaptation strategies towards climate change-induced natural disasters. Training (30.1%), national radio (27.7%), and television (19.1%) were the preferred leading methods for receipt of global warming information. These findings provide useful insights for policy directions. The government of Ghana and other stakeholders should develop a communication strategy to increase and sustain publicity and education on climate change to the citizenry.

## 1. Introduction

The adverse impact that climate change has on the health and lives of billions of people is of great concern to the world. It is expected that climate change will cause an estimated 250,000 deaths per year between 2030 and 2050 as a result of malnutrition, malaria, diarrhoea, and heat stress, with direct damage costs to the health of between USD 2–4 billion/year by 2030 [1]. Climate change affects human health in several ways. The direct effects of climate change hazards such as extreme weather, storms, rising sea levels, flooding, and drought threaten health and life [2] and create enabling conditions that result in increased transmission of infectious diseases [3–6]. Climate change also triggers events like migration, displacement, and conflicts which have adverse health implications on humans [7, 8].

The consequences of climate change are experienced in several ways by countries within the sub-Saharan African (SSA) region. Climate change projections within SSA include warming inland subtropics, extreme heat occurrences, increased dryness, and change in rainfall patterns, more especially in Southern and Eastern Africa [9]. As a result of climate change, the already high rates of undernutrition and infectious diseases in SSA are expected to increase. Agricultural systems, which serve as sources of livelihoods for a large proportion of the region's population, are vulnerable to climate change [10]. Livelihoods are destroyed, and rural-urban migration increases as a result of climate change, leading to a chain of events like disease outbreaks, increased food prices, and conflicts that exacerbate the worse economic conditions in SSA [9, 11].

Climate change aggravates conditions that lead to health inequities and inequalities for vulnerable populations in SSA. The impacts of climate change worsen social determinants like poverty, educational level, and food security, which play a significant role in determining the extent of health [12, 13]. This situation poses an increased health risk to populations that are already vulnerable, resulting in increased health inequity. The health impact of climate change calls for effective mitigation and adaptation measures. Recommended measures to address the health impacts of climate change on populations and health systems include vulnerability assessment of populations and geographical areas, as well as the capacity of health systems to manage such vulnerabilities, also known as vulnerability and adaptation assessment [14]. Even though such assessments are beneficial, the populations affected by climate change must be aware of the array of issues surrounding it. Populations need to understand the climate change ecosystem in order to contribute to the numerous awareness, education, adaptation, and mitigation measures. Although it may be unrealistic to expect the ordinary person to think about climate change like atmospheric scientists or policy analysts, this represents an essential element concerning effective policymaking, and it is, therefore, necessary to understand “where the public stands” on this issue. For people, to adapt effectively to climate change, they must have correct perceptions about the state of the climate and possible future trends [15] and impact. These perceptions inform recommendations on the appropriate strategies of collaborating and communicating with the public on climate change.

A considerable number of studies have examined the influences on people's attitudes and behaviour relevant to climate change [16–19]. Although these studies have been useful in helping raise global awareness on the topic, serious gaps remain in the understanding of attitudes and behaviours concerning climate change, particularly in sub-Saharan Africa, including Ghana due to paucity of research. Thus, there is little or nothing the typical Ghanaian can do to respond to the impact of climate change. Nevertheless, to have the highest chance to slow and, perhaps, even reverse the slide towards calamitous climate change, we need to mobilize the broadest possible public support for practical actions [20, 21]. For this to be done effectively, there is the need to better understand the knowledge and perceptions, as well as the attitudes and behaviours of the Ghanaian populace towards climate change.

This study aimed to assess the level of knowledge and adaptation to climate change amongst people in Ghana. To achieve our objective, we sought to answer four main questions: (1) What is the level of knowledge and perceptions of climate change amongst Ghanaians? (2) How do Ghanaians explain the causes of their changing climate and subsequent effects on their lives? (3) What are the best methods of communicating climate change and related issues to the Ghanaian populace? (4) What are the implications of the data obtained and their input for policy design? Answers to these research questions can be used by the government and other stakeholders to understand the level of knowledge and awareness of the concept of climate change amongst Ghanaians and to craft policies and programmes aimed at promoting successful adaptation, mitigation, and disaster risk management.

## 2. Methodology

### 2.1. Research Design

The study employed a descriptive, cross-sectional design with self-administered questionnaires to assess the level of knowledge, attitudes, and adaptation to climate change in Ghana. The study was conducted from August 1, 2019, to December 31, 2019. Questionnaires were self-administered and took an average of 25 minutes to complete. The average margin of error (95% confidence interval) for the survey was three percentage points.

### 2.2. Survey Subjects and Technique

Data were obtained from a nationally representative survey of Ghanaian adults (*N* = 674). The study utilized a stratified sampling technique. The country was demarcated into three zones: southern belt, middle belt, and northern belt. Therefore, in selecting the respondents for the survey, a sampling proportionate to size was utilized to determine the number of respondents to be interviewed from each zone. All adults 18 years above present in the demarcated zone were considered for the study.

### 2.3. Survey Content

The study employed a standardized structured questionnaire designed to achieve the aims of the research for data collection. The constructs in the questionnaires were informed by literature on climate change and its effects. After each day's interviews, field inspection of questionnaire data was done, allowing for immediate verification and correction of errors that were identified. The final survey instrument comprised 76 questions in nine thematic areas: sociodemographics (7 items), knowledge and understanding of climate change (8 items), attitude towards climate change (items 5), an encounter with natural disasters (9 items), perceived causes of climate change (7 items), perception of the future effects of climate in Ghana (10 items), feelings towards climate change (9 items), adaptation strategies against natural disasters encountered (13 items), and preferred methods to receive information on climate change warming (8 items).

Six experts in social sciences measurement and evaluation determined the face validity of the instrument. The average overall face validity was equal to 95%. The study used Cronbach's alpha test for reliability testing, which yielded a reliability coefficient of 0.8. Cronbach's alpha test assessed the internal consistency of a set of the scale of items to ensure that they were all consistent in measuring the same attributes under study.

### 2.4. Ethical Consideration

Ethical clearance was obtained from the Ethics Review Committee (ERC) of the GIMPA School of Public Service and Governance. Verbal consent and written consent were obtained from respondents before the data collection instruments were distributed. It was ensured that enough information related to the aims of the study was given to the respondents. Respondents were reassured of confidentiality and were duly informed that participation in the study was voluntary, and they were free to decline participation at any time.

### 2.5. Statistical Analysis

The data obtained from the study were verified, coded, and subsequently analysed using SPSS version 23. Appropriate text statistics were performed. These include Chi-Squared (*X*^2^) tests. Two-tailed statistical testing was also used. An alpha value of 0.05 or below was considered significant for all statistical tests.

## 3. Results

### 3.1. Demographic Data of Respondents

Table 1 presents the demographic data of the respondents. A total of 674 respondents participated in this study. This cohort included 353 females and 321 males representing 52.4% and 47.6%, respectively. Almost half of the respondents (47.3%) were in the age of 18–30 years group, followed by 31–48 years (32.3%), and the least (7.6%) were 68 years and above. The majority of the respondents (79.7%) were Christians, followed by Muslims (19.4%) and Traditionalists (0.9%). In relation to respondents' ethnic group, most of the respondents were Akans (41.5%), followed by Ga-Adangbes (25.4%), Ewes (20.9%), and the least being Mole-Dagbons (2.8%). Most of the respondents were single (71.8%), and the rest were married (22.3%), widowed (3.7%), and divorced (2.2%).

More than half of the respondents (57.4%) had undergraduate education, and the rest had secondary education (22.7%), postgraduate (9.1%), no formal education (5.6%), and primary education (4.9%). When asked to rate their social status, the majority of the respondents (63.2%) said that they were in the middle class, followed by the upper class (29.7%) and lower class (7.1%). Almost all the respondents (89.2%) were living in urban areas whilst 10.8% were living in rural areas.

**Figure 1:**
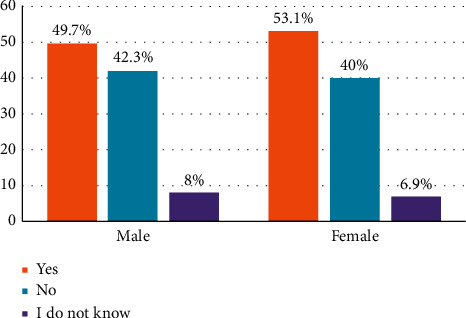
Respondents' encounter with natural disasters.

Figure 1 shows the respondents' encounter with a natural disaster. There was an almost equal response (50 : 50) from the respondents when asked if they encountered any natural disasters. We observed that 53.1% of the females indicated that they had encountered natural disasters as compared to males (49.7%). Furthermore, 41% of the males indicated that they had not encountered any natural disaster, whilst 40% of the females responded the same.

Table 2 shows the respondents' general understanding of climate change. When questioned on their understanding of climate change, 481 (71.4%) stated that they had heard of climate change and 296 (43.9%) indicated that they understood the causes and impacts of climate change. However, 20.8% of the respondents had not heard of climate change, and 7.9% did not know of climate change before this interview was conducted. In terms of understanding, 35.2% of the respondents indicated that they understood climate change to some extent, and 3.9% did not understand climate change at all.

Table 3 presents assessments of respondents' perceptions of the causes of climate change. Burning of fossil fuel (54%), carbon emission from vehicles (17.5%), and deforestation (17.1%) were the top three indicators by the respondents.

Table 4 shows the respondents' perception of the future effects of climate change in Ghana. The majority of the respondents (74.5%) were able to indicate at least one future effect of climate change, as against 19.1% who indicated that they did not know of any future effect and 6.4% indicating that there would be no future effect of climate change in Ghana.

Table 5 shows how the respondents felt about climate change. The majority of the respondents (32.8%) indicated that they became afraid every time they heard of climate change issues on televisions, radios, and social media platforms or discussed by other people. Comparatively, 11.6% of the respondents became sad because of the frequent floods which have destroyed lives and properties and rendered other people homeless in other communities and nations, and 10.7% reported that they were confused about climate change. However, 10.8% stated that they had no feelings towards climate change since they had no idea of its consequences, and 13.6% were hopeful that something could be done to adapt to climate change.

The attitudes of the respondents towards climate change are shown in Table 6. Most of the respondents (45.1%) strongly agreed and 39.2% agreed that climate change was occurring due to frequent rainfall and temperature rise in their communities over the years. A higher percentage of respondents (39.2%) agreed and 34.1% strongly agreed that human activity was responsible for climate change, but 1.5% strongly disagreed with this assertion. Most of the respondents agreed or strongly agreed (34.4% and 23.6%) that natural changes in the environment were responsible for climate change whilst 2.7% disagreed. Moreover, 22% of respondents agreed whilst an almost equal number (23.3%) strongly disagreed that living for today was more important than worrying about the effects of climate change in fifty years. The majority of the respondents (37.5%) agreed whilst 2.2% strongly disagreed that climate change could reduce the quality of life for future generations.

Action taken or otherwise to adapt to climate change is shown in Table 7. The majority of the respondents (50.1%) stated that they were doing something to adapt to climate change followed by 34.6% who were not acting to adapt to climate change and 15.3% who did not know what to do to adapt to climate change.

**Figure 2:**
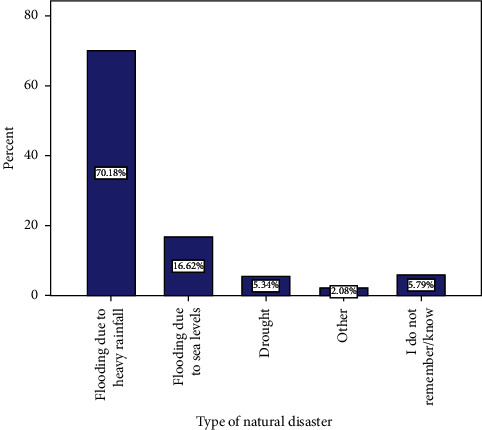
Type of natural disasters encountered by respondents.

Figure 2 depicts the various types of natural disasters encountered by the respondents. When respondents were asked about the type of natural disasters they had encountered, 70.18% stated flooding due to heavy rainfall, 16.62% stated flooding due to sea level, 5.34% stated drought, 5.79% stated they did not know or could not recollect any natural disaster encountered in their community, and 2.08% reported other natural disasters.

Table 8 shows respondents' adaptation strategies against natural disasters that they encountered. When the respondents were asked to select all that applied to them from a list of strategies to prevent or minimise the effect of natural disasters encountered, the following results were obtained: 30.2% of the respondents indicated that they distilled or maintained public drainage from waste; 25.6% indicated that they cleared drains; 13.5% indicated that they made their properties more resistant; 11.9% maintained trees and vegetation to absorb excess water from severe rainfall and reduce the surface temperature in order to reduce drought; 5.5% built wells and/other water resources. Some respondents (9.6%) also indicated that they did nothing or did not know what to do to prevent or minimise the effect of natural disasters they encountered.

Table 9 shows guidance and training on climate change received by respondents. Of the respondents, 50.7% indicated that they had been told by someone in authority what to do if a disaster such as flooding or droughts occur while 45.5% of the respondents indicated that they have no information on what to do if a disaster such as flooding or droughts occurs. The majority of the respondents (48.7%) stated that they had never attended a consultation meeting, seminar or workshop, or school lesson on climate change whilst 44.5% stated that they had ever attended an event on climate change.

The preferred method of receiving information on climate change, as indicated by the respondents, is shown in Table 10. A high percentage of respondents (30.1%) stated that they preferred to receive climate information in their schools or through training programmes. Twenty-seven (27.7%) prefer national radio, 27.2% prefer televisions, and 7.3% prefer social media.

## 4. Discussion

The study aimed to assess the level of knowledge and adaptation to climate change among people in Ghana. The majority of the study respondents had some form of formal education. Almost one out of ten respondents (9.1%) had postgraduate education, 57.4% had undergraduate education, 22.7% had secondary education, and 4.9% had primary education. It is encouraging that most of the respondents in this study have had formal education (94.1%). Several studies highlight the vital role that formal education plays in increasing people's capacity to adapt to climate change [22–24]. Ghana can take advantage of its high 86% youth literacy rate [25] to promote and implement adaptive strategies to climate change. As asserted by Smith and Leiserowitz [26], one vital strategy that may prove to address challenges related to climate change is targeting climate literacy efforts towards young people.

A high percentage of the respondents had heard about climate change (71.4%). In contrast, a higher percentage did not have a clear understanding of what climate change entailed. Only 43.9% indicated that they had a complete understanding of climate change, and 11.8% did not understand climate change at all. Even though hearing about climate change is an initial step to start a climate change conversation, it is of concern that the percentage of respondents who understood climate and its effects was lower than those who have heard of climate change. This finding might be attributed to the less public awareness campaigns on climate change issues to the general public, in both Ghana and Africa in general [27]. The low knowledge of climate change among participants should be of great concern because it contributes to the acceptance of global warming, as reported in a study by Stevenson, Peterson, Bondell, Moore, and Carrier [28]. In their study, Stevenson et al. [28] found that increased knowledge of climate change was positively associated with the acceptance of global warming. These results highlight the need to address low climate literacy among the Ghanaian population.

The study also assessed the perception of respondents on what causes climate change leading to natural disasters. The study respondents attributed the cause of climate change over the years to natural processes and human activities. The majority of the respondents (54.2%) indicated the burning of fossil fuel such as oil and coal as the cause of climate change, followed by carbon emission from vehicles (17.5%), deforestation (17.1%), and natural events such as ocean currents (5.3%). A low percentage of respondents (2.5%) indicated agricultural emissions of nitrous oxide from fertilizers as a cause of climate change. This low percentage is an indication that most Ghanaians do not have much knowledge about the fact that livestock contributes to about 14.5% of anthropogenic emissions of the total greenhouse gas emissions [29].

The amount of knowledge on climate change influences the risk perception of individuals. It was interesting to note that some of the respondents (1.5%) attributed the cause of climate change to God. This may be due to the high religious beliefs of Ghanaians [30]. When asked about the future effects of climate change, the majority of the respondents (74.5%) were able to identify at least one future effect of climate change, as against 19.1% indicating that they did not know of any future effect and 6.4% indicating that there would be no future effect of climate change in Ghana. It is of utmost concern that about 25% of the participants did not have much knowledge about the impacts of climate change as well as future consequences. Knowing what causes climate change and its effects is a significant determinant of people's intention to take voluntary precautionary measures and influence climate change policies [31]. Previous research by Smith and Leiserowitz [26] also suggests that the perception of climate change risks is associated with the available knowledge.

From the analysis, close to half of the respondents (49.2%) do not have a clue as to what to do in the case of a disaster resulting from climate change even though the majority have participated in climate change activities. This finding depicts that the general public is at high risk should any natural disaster occur. Many people in Ghana will not know what to do when extreme weather conditions arise from climate change. The knowledge of people affected by disasters has an impact on their participative behaviours, which plays a critical role in complementing systems that are built in response to natural disasters [32]. It is, therefore, imperative to build adequate local resilience for emergency response when disaster strikes in Ghana.

The percentage of participants that have participated in climate change activity (44.5%) is an indication that, even though minimal, some efforts are being made towards communicating climate change and its related issues in Ghana. This signal is in line with the assertion by the Ghana National Climate Change Policy that organisations and development partners are contributing to Ghana's response towards addressing climate change [33]. However, there is the need to scale up these efforts to mainstream climate change into national development strategies.

Adaptation and resilience strategies are essential to combat the effects of climate change. However, in our study (Table 8), we found that the primary means employed by the respondents to combat natural disasters they encountered was to distil or maintain public drainage systems. This strategy suggests attempts to prevent flooding. Flooding is the leading natural disaster and environmental challenges faced by many countries, including Ghana. [34–36]. In Ghana, flooding appears to be the manifestation of the changing climate patterns. It presents with a dire consequence, including disruption of socioeconomic activities, extensive damage to property, infrastructure, and communication facilities, and loss of life [37]. It also burdens the already weak health systems as it causes outbreaks of gastroenteritis, communicable diseases, epidemic diseases such as cholera, diarrhoea, among others [34–36, 38–40].

In terms of information dissemination, the respondents were of the view that training has been shown to be an effective tool to be employed in providing information on climate change. The majority of participants preferred to receive climate change information through school training. A large proportion of the participants (54.9%) also preferred to receive information on climate change via television and radio. Information on climate change has a high probability of being trusted if they are done through these channels. From the data, there is an indication that the traditional methods of disseminating information could be employed to create awareness on climate change. The popularity and familiarity of these tools will facilitate the dissemination of climate change information. However, there is the need to drive the agenda to promote the science behind climate change in the media in order to reduce the influence that worldviews have on how people perceive the risks associated with climate change. Smith and Leiserowitz [26] assert that efforts to communicate climate change risks may be ineffective because “worldview rather than scientific understanding largely drives climate change risk perceptions.”

### 4.1. Study Limitations

Since this is a cross-sectional study, this study has limitations of a cross-sectional study. The results observed could be different if measured at another time. Other study limitations include self-selection and self-reports and those who refused to participate in the study.

Furthermore, a very high proportion of the study participants (66.8%) were college educated and had at least a bachelor's degree and 22.7% had secondary or high school education. It may be argued that these highly educated individuals, that is, two out of every three respondents, might have introduced bias into the study. This educational bias should have worked for us but it rather worked against us; hence, 20.8% of the respondents had not heard of climate change, and 7I.9% did not know of climate change before this interview was conducted. Furthermore, a high percentage of the respondents had heard about climate change 71.4% which should probably have been 89.5% if education was a proxy for hearing about climate change. In addition, only 43.9% indicated that they had a complete understanding of climate change, and 11.8% did not understand climate change at all. Thus, it can be noted that if bias existed at all because of the sampling, it was negligible from the results we obtained.

## 5. Conclusion and Policy Implication

The study examined the public knowledge of respondents about climate change in Ghana. The study undoubtedly revealed useful insights for policy directions in Ghana. Results from the research showed that the majority of the Ghanaians who participated in the study had low awareness of climate change even though formal education was high among them. Based on these and other results, several useful points for policy in this regard are outlined.

First, the relevant state agencies and all other significant stakeholders should join forces to increase publicity and education about climate change and its effects. Government need to take advantage of the high level of formal education among its youth to create awareness about climate change. Strategies to increase climate change awareness should be targeted towards the youth because they have a significant stake in the future. Policies and strategies should be crafted with an understanding of youth dissent in order to engage young people in diverse ways to promote climate-resilient futures [41].

Secondly, policies to address climate change in Ghana should be aimed at improving the understanding that people have about the causes of climate change. The results of a study conducted by Shi, Visschers, Siegrist, and Arvai [42] suggested that people were more likely to be concerned about climate change if their knowledge of the underlying causes of climate change was high. It is imperative that Ghana crafts its climate change policies and activities towards improving the knowledge of the population about climate change causes rather than the physical characteristics of climate change. Having a better understanding of the causes of climate change will trigger behavioural actions among the Ghanaian populace to reduce human activities that contribute to climate change.

Finally, with regard to the causes of climate change, certain misconceptions are still lingering. A significant number of respondents indicated that global warming was an act by God. Also, most participants were either undecided or disagreed that climate change could reduce the quality of life for future generations. Hence, stakeholders and policymakers should strongly emphasise sustainability when making decisions and policies on climate change awareness creation.

In conclusion, it is worth noting that educating the citizenry on the effects and adaptation strategies of climate change will require a deepened public engagement of all stakeholders. It will provide a handy tool by which the development of effective public policies as well as citizenry participation can occur.

## Figures and Tables

**Table 1:** Demographic table.

Variable	Female *N* (%)	Male *N* (%)	Total *N* (%)	Significant value
**Age**				
18–30	191 (28.3)	128 (19.0)	319 (47.3)	*X* = 15.667
31–48	93 (13.8)	124 (18.4)	217 (32.2)	*P*=0.001
49–68	42 (6.2)	45 (6.7)	87 (12.9)	
68 and above	27 (4.0)	24 (3.6)	51 (7.6)	
Total	353 (52.4)	321 (47.6)	674 (100)	

**Religion**				
Christianity	285 (42.3)	252 (37.4)	537 (79.7)	*X* = 6.715
Islamic	68 (10.1)	63 (9.3)	131 (19.4)	*P*=0.035
Traditionalist	0 (0.0)	6 (0.9)	6 (0.9)	
Total	353 (52.4)	321 (47.6)	674 (100)	

**Ethnicity**				
Akan	155 (23.0)	125 (18.5)	280 (41.5)	*X* = 11.298
Ga-Adangbe	101 (15.0)	70 (10.4)	171 (25.4)	*P*=0.023
Mole-Dagbon	9 (1.3)	10 (1.5)	19 (2.8)	
Ewe	60 (8.9)	81 (12.0)	141 (20.9)	
Other	28 (4.2)	35 (5.2)	63 (9.3)	
Total	353 (52.4)	321 (47.6)	674 (100)	

**Marital status**				
Single	266 (39.5)	218 (32.3)	484 (71.8)	*X* = 29.895
Married	56 (8.3)	94 (13.9)	150 (22.3)	*P* ≤ 0.001
Divorced	15 (2.2)	0 (0.0)	15 (2.2)	
Widow/widower	16 (2.4)	9 (1.3)	25 (3.7)	
Total	353 (52.4)	321 (47.6)	674 (100)	

**Highest education**				
No formal education	24 (3.6)	14 (2.1)	38 (5.6)	*X* = 24.457
Primary	21 (3.1)	12 (1.8)	33 (4.)	*P* ≤ 0.001
Secondary	57 (8.5)	96 (14.2)	153 (22.7)	
Undergraduate	225 (33.4)	164 (24.3)	389 (57.7)	
Postgraduate	26 (3.9)	35 (5.2)	61 (9.1)	
Total	353 (52.4)	321 (47.6)	674 (100)	

**Social status**				
Upper class	123 (18.2)	77 (11.4)	200 (29.7)	*X* = 12.097
Middle class	212 (31.5)	214 (31.8)	426 (63.2)	*P*=0.002
Lower class	18 (2.7)	30 (4.5)	48 (7.1)	
Total	353 (52.4)	321 (47.6)	674 (100)	

**Residence**				
Rural	35 (5.2)	38 (5.6)	73 (10.8)	*X* = 0.644
Urban	318 (47.2)	283 (42.0)	601 (89.2)	*P*=0.422
Total	353 (52.4)	321 (47.6)	674 (100)	

**Table 2:** Respondents' understanding of climate change.

Gender	Heard of climate			Understanding of climate change				
	Yes *N* (%)	No *N* (%)	Don't Know *N* (%)	Yes *N* (%)	To some extent *N* (%)	Not really *N* (%)	No *N* (%)	I do not know *N* (%)
Female	265 (39.3)	99 (9.8)	22 (3.8)	124 (18.4)	144 (21.4)	32 (4.7)	18 (2.7)	35 (5.2)
Male	216 (32.0)	74 (11.0)	31 (4.6)	172 (25.5)	93 (13.8)	30 (4.5)	8 (1.2)	18 (2.7)
**Total**	481 (71.4)	140 (20.8)	53 (7.9)	296 (43.9)	237 (35.2)	62 (9.2)	26 (3.9)	53 (7.9)

**Table 3:** Respondents' perceptions of climate change causes.

Cause	*N* (%)
Burning fossil fuel	365 (54.2)
Deforestation	115 (17.1)
Natural events such as ocean currents	36 (5.3)
Carbon emission from vehicles and industries	118 (17.5)
Agricultural emissions of nitrous oxide from fertilizers	17 (2.5)
Acts of God	10 (1.5)
Others	13 (1.9)
**Total**	674 (100)

**Table 4:** Respondents' perception of the future effects of climate in Ghana.

Future effects of climate change	Female *N* (%)	Male *N* (%)	Total *N* (%)
More rain	45 (6.7)	71 (10.5)	116 (17.2)
Rise in sea level	57 (8.5)	55 (8.2)	112 (16.6)
Increase in erosion	16 (2.4)	23 (3.4)	39 (5.8)
Less rain	41 (6.1)	35 (5.2)	76 (11.3)
Hotter temperature	51 (7.6)	58 (8.6)	109 (16.2)
Colder temperature	8 (1.2)	0 (0.0)	8 (1.2)
Trees may die	14 (2.1)	6 (0.9)	20 (3.0)
Ocean temperature	10 (1.5)	12 (1.8)	22 (3.3)
I do not know	82 (12.2)	47 (7.0)	129 (19.1)
No effect	29 (4.3)	14 (2.1)	43 (6.4)
**Total**	353 (52.4)	321 (47.6)	674 (100.)

**Table 5:** Respondents' feelings towards climate change.

How do you feel about climate change?	Female *N* (%)	Male *N* (%)	Total *N* (%)	Significant value
Fearful/afraid	103 (15.3)	118 (17.5)	221 (32.8)	*X* = 42.265
Confused	45 (6.7)	27 (4.0)	72 (10.7)	*P* ≤ 0.001
Angry	0 (0.0)	13 (1.9)	13 (1.9)	
Sad	37 (5.5)	41 (6.1)	78 (11.6)	
Powerless	30 (4.5)	25 (3.7)	55 (8.2)	
No feelings	45 (6.7)	28 (4.2)	73 (10.8)	
Hopeful	65 (9.6)	27 (4.0)	92 (13.6)	
Do not believe it exists	8 (1.2)	6 (0.9)	14 (2.1)	
I do not know	20 (3.0)	36 (5.3)	56 (8.3)	
**Total**	353 (52.4)	321 (47.6)	674 (100)	

**Table 6:** Respondents' attitude towards climate change.

Statement	Strongly agree *N* (%)	Agree *N* (%)	Undecided *N* (%)	Disagree *N* (%)	Strongly disagree *N* (%)
Climate change is occurring	304 (45.1)	235 (34.9)	135 (20.0)	0 (0)	0 (0)
Human activity is responsible for climate change	230 (34.1)	264 (39.2)	170 (25.2)	0 (0)	10 (1.5)
Natural changes in the environment are responsible for climate change	159 (23.6)	232 (34.4)	192 (28.5)	73 (10.8)	18 (2.7)
Living for today is more important than worrying about the effects of climate change in fifty years	121 (18.0)	148 (22.0)	146 (21.7)	102 (15.1)	157 (23.3)
Climate change can reduce the quality of life for future generations	248 (36.8)	253 (37.5)	140 (20.8)	18 (2.7)	15 (2.2)

**Table 7:** Action taken to adapt to climate change.

Variable	Female *N* (%)	Male *N* (%)	Total	Significant value
Yes	202 (30.0)	136 (20.2)	338 (50.1)	*X* = 25.394
No	91 (13.5)	142 (21.1)	233 (34.6)	*P* ≤ 0.001
I do not know	60 (8.9)	43 (6.4)	103 (15.3)	
**Total**	353 (52.4)	321 (47.6)	674 (100)	

**Table 8:** Respondents' adaptation strategies against natural disasters encountered.

Variable	*N*	%
Made property more resistant to threats	91	13.5
Cleared drains	173	25.6
Maintained trees and vegetation	79	11.9
Built wells and/other water resources	38	5.6
Distilled or maintained public drainage from waste	204	30.2
Did nothing	65	9.6
I do not know	16	2.4
Other	8	1.2

**Table 9:** Guidance and training on climate change disasters.

Question	Yes *N* (%)	No *N* (%)	I do not know *N* (%)
Have you been told by someone in authority what to do if a disaster such as flooding or droughts occurs?	342 (50.7)	307 (45.5)	25 (3.7)
Have you ever attended a consultation meeting, seminar or workshop, or school lesson on climate change?	300 (44.5)	328 (48.7)	46 (6.8)

**Table 10:** Preferred method of receiving climate information.

	Female *N* (%)	Male *N* (%)	Total *N* (%)
National radio	72 (10.7)	115 (17.1)	187 (27.7)
School/training	119 (17.7)	84 (12.5)	203 (30.1)
TV	91 (13.5)	92 (13.6)	183 (27.20)
Local newspaper	0 (0.0)	6 (0.9)	6 (0.9)
Social media	39 (5.8)	10 (1.5)	49 (7.3)
Billboards/posters	3 (0.4)	0 (0.0)	3 (0.4)
None	26 (3.9)	14 (2.1)	40 (5.9)
Others	3 (0.4)	0 (0.0)	3 (0.4)
**Total**	**353 (52.4)**	**321 (47.6)**	**674 (100)**

## Data Availability

The data used to support the findings of this study are available from the corresponding author upon request.
